# Eco-Friendly Membrane Separators Based on Furanoate Polymers for Li-Ion Batteries

**DOI:** 10.3390/polym17202790

**Published:** 2025-10-18

**Authors:** Sofia Santi, Luca Bargnesi, Giulia Fredi, Michelina Soccio, Nadia Lotti, Catia Arbizzani, Andrea Dorigato

**Affiliations:** 1 INSTM Research Unit, Department of Industrial Engineering, University of Trento, Via Sommarive 9, 38123 Trento, Italy; 2 Department of Chemistry “Giacomo Ciamician”, University of Bologna, Via Gobetti 85, 40129 Bologna, Italycatia.arbizzani@unibo.it (C.A.); 3 Department of Civil, Chemical, Environmental, and Materials Engineering, University of Bologna, Via Terracini 28, 40131 Bologna, Italy; m.soccio@unibo.it (M.S.);; 4 Interdepartmental Center for Industrial Research on Advanced Applications in Mechanical Engineering and Materials Technology (CIRI-MAM), University of Bologna, 40100 Bologna, Italy; 5 Interdepartmental Center for Agro-Food Research (CIRI-AGRO), University of Bologna, 40100 Bologna, Italy

**Keywords:** biopolymers, polyesters, furanoates, Li batteries, electrospinning

## Abstract

Conventional lithium-ion battery separators made from petroleum-based polymers pose environmental concerns due to their non-renewable origin and energy-intensive production. Novel bio-based alternatives, such as poly(alkylene 2,5-furanoate)s (PAFs), offer improved sustainability and favorable thermomechanical properties. This work investigated electrospun mats of poly(butylene 2,5-furandicarboxylate) (PBF) and poly(pentamethylene 2,5-furandicarboxylate) (PPeF), which, despite structural similarity, exhibit distinct behaviors. PBF mats demonstrated superior performance with fiber diameters of about 1.0 µm and porosity of 53.6% with high thermal stability (T_g_ = 25 °C, T_m_ = 170 °C, 18.8% crystallinity). The semicrystalline PBF showed higher electrolyte uptake (531–658 wt%) and had a lower MacMullin number (*N_M_* = 3–10) than commercial Celgard separators (*N_M_* = 15), indicating enhanced ionic conductivity. Electrochemical testing revealed stability up to 5 V and successful cycling performance with specific capacity of 135 mAh/g after 100 cycles and coulombic efficiency near 100%. In contrast, PPeF’s amorphous nature (T_g_ = 14 °C) resulted in temperature-sensitive pore closure that enhanced safety by reducing short-circuit risk, although its solubility in carbonate electrolytes limited its application to aqueous systems. These findings highlight the potential of PAF-based separators to improve both the environmental impact and performance of batteries, supporting the development of safer and more sustainable energy storage systems.

## 1. Introduction

Lithium-ion batteries (LIBs) have been extensively researched and have found widespread use in various practical applications, including electronic devices and electric vehicles. Recently, sodium-ion batteries (SIBs) have also gained traction in commercial applications. However, as LIBs and SIBs are increasingly deployed in a wide range of applications, battery safety has become a critical challenge to overcome. Despite the benefits of LIBs, severe electric accidents due to battery module damage have raised concerns about the safety of LIBs. The separator plays a crucial role in the functioning of both LIBs and SIBs, directly impacting battery safety. It serves as a physical barrier that prevents direct contact between the cathode and anode while holding the electrolyte to facilitate the movement of ions within the battery. Its failure can pose serious safety risks. Common failure mechanisms include (i) high environmental temperature causing the separator to melt, shrink, or even ignite; (ii) separator damage due to collisions or pressure, leading to direct contact between the anode and cathode; (iii) penetration of the separator by metal dendrites formed during overcharging, resulting in an internal short circuit [1]. Thus, high-safety separators must possess excellent heat resistance to prevent shrinkage at elevated temperatures and potentially flame-retardant properties; strong resistance to dendrite growth; high mechanical strength to maintain structural integrity, whether as an independent component or integrated within the battery; and superior chemical compatibility to minimize reactions with other battery components and ensure good affinity with the electrolyte.

Traditionally, the membrane separators in LIBs are made from synthetic polymers, such as polyethylene (PE) or polypropylene (PP), which are derived from non-renewable petroleum resources [2,3]. These materials, while effective in terms of their performance, present environmental concerns due to their origin and the energy-intensive processes required for their production. In response to these challenges, there has been growing interest in the development and implementation of sustainable and eco-friendly membrane separators [4,5,6,7]. These separators, derived from renewable resources, offer a promising alternative to conventional materials. Sustainable membrane separators can significantly reduce the environmental footprint of LIBs, aligning with the broader goals of green energy and circular economy initiatives.

Among bio-based polymers, poly(alkylene 2,5-furanoate)s (PAFs) are particularly noteworthy. Synthesized from 2,5-furandicarboxylic acid (FDCA) and glycols of varying alkyl chain lengths, PAFs demonstrate superior thermal stability and thermomechanical properties compared to their petroleum-derived counterparts [8,9,10]. Poly(ethylene 2,5-furandicarboxylate) (PEF) is a well-studied furan-based polyester and a promising bio-based alternative to poly(ethylene terephthalate) (PET) in packaging, textiles, and medical applications [9]. More recently, other furanoate polyesters with longer alkyl chains, such as poly(butylene 2,5-furandicarboxylate) (PBF) [11,12,13,14] and poly(pentamethylene 2,5-furandicarboxylate) (PPeF) [15,16], have gained attention for a variety of uses. Structurally, these two polymers differ by a methyl group in the alkyl chain of their repeat units. However, despite their structural similarities, they exhibit significantly different physical, thermal, mechanical, and biological properties. PBF is semicrystalline and exhibits thermomechanical characteristics similar to those of poly(butylene terephthalate) (PBT) but with a lower processing temperature and greater ductility, featuring a glass transition temperature (T_g_) of 33–35 °C and a melting point (T_m_) of around 172 °C. In contrast, PPeF is generally amorphous and incorporates a diol with five carbon atoms, influenced mainly by the odd–even effect, where the C-odd-numbered glycolic subunits decrease the crystallization tendency and significantly alter the thermomechanical properties [17,18,19,20]. This results in a lower T_g_ (17 °C), greater ductility, and increased strain at break [21,22].

The feasibility of producing nanofibrous mats of PBF and PPeF through electrospinning has been systematically explored in previous works by our group [19,20], demonstrating the tunability achieved by producing fibers of varying diameters and optimizing the formation of electrospun mats with homogeneous porosity, useful for electrolyte transition and maintaining separation between the anode and cathode. The electrospun mats of PBF displayed good geometric stability from −50 °C to 250 °C thanks to the crystalline domains, while the low T_g_ of the amorphous PPeF led to the closing of pores above 14 °C.

For these reasons, the present work explores the advantages of using sustainable and eco-friendly membrane separators based on PBF and PPeF in lithium-ion batteries by examining their physicochemical properties and electrochemical behavior to highlight the role these materials play in advancing the sustainability of energy storage systems.

## 2. Materials and Methods

### 2.1. Materials

The materials used in this work were dimethyl 2,5-furandicarboxylate (2,5-DMF, SarchemLabs, Farmingdale, NJ, USA, CAS: 4282-32-0), 1,4-butanediol (1,4-BD, Sigma Aldrich, St. Louis, MI, USA, 99%pur, CAS: 110-63-4), 1,5-pentanediol (1,5-PD, Fluka, Honey well International Inc., Charlotte, NC, USA, ~97% purity, CAS: 111-29-5), hexafluoro-2 propanol (HFIP, CarloErba, Cornaredo, Italy, CAS: 920-66-1), chloroform (CHCl_3_, Carlo Erba, analytical standard purity of 99.5%, CAS: 67-66-3,), dimethylformamide (DMF, Sigma Aldrich, 99.5% purity, CAS: 68-12-2), ethanol (Sigma Aldrich, Merck Life Science S.r.l., Milan, Italy, absolute ≥ 99.8%, CAS: 64-17-5), polytetrafluoroethylene from aqueous suspension (PTFE, Dupont De Nemour Netherlands B.V., Dordrecht, Netherlands, 60 wt/w%), conductive additive (Super C45, Imerys Graphite & Carbon Switzerland SA, Bodio, Switzerland), activated carbon (Picactif BP10 PICA USA, Inc., Columbus, OH, USA), lithium iron phosphate (LFP) electrode tapes (NEI Corporation, Somerset, NJ, USA), 1 M LiPF_6_ in ethylene carbonate: dimethyl carbonate (EC:DMC) 1:1 wt% (LP30, Solvionic, battery grade, 99.9%, Toulouse, France).

### 2.2. Sample Preparation

**Figure 1:**
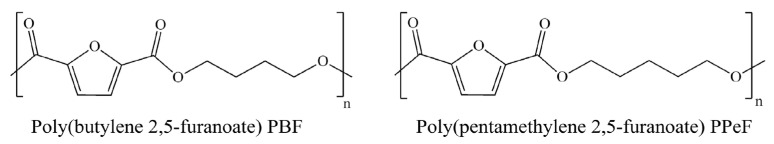
Chemical structures of PBF and PPeF.

**Synthesis of PBF and PPeF matrices.** Poly(butylene 2,5-furanoate) (PBF) and poly(pentamethylene 2,5-furanoate) (PPeF) were synthesized at the laboratory scale through a solvent-free polycondensation process, according to the procedure described in Guidotti et al. [23], starting from dimethyl 2,5-furandicarboxylate (2,5-DMF) and glycols, 1,4-butanediol (1,4-BD) for PBF and 1,5-pentanediol (1,5-PD) for PPeF. Titanium tetrabutoxide (TBT) and titanium isopropoxide (TIP) were used as catalysts. The chemical structures of PBF (average molecular weight M_n_ = 36,500 g/mol) and PPeF (M_n_ = 43,900 g/mol) are reported in Figure 1.

**Figure 2:**
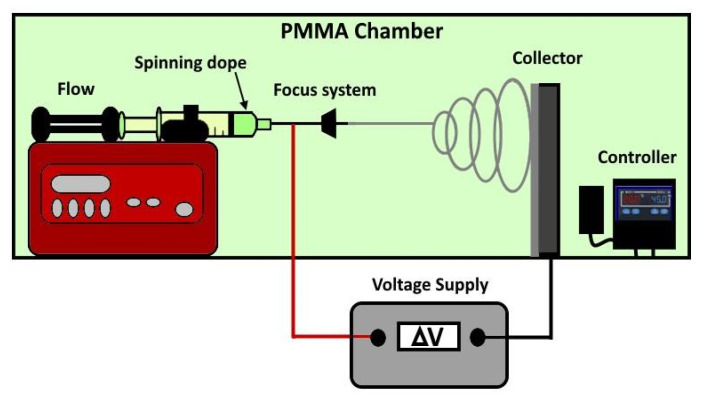
Schematic representation of the laboratory-scale electrospinning setup (duplicated from [20]).

**Preparation of PBF and PPeF nonwoven mats.** The electrospinning setup, reported in Figure 2, was confined in a polymethylmethacrylate (PMMA) chamber with monitored temperature and humidity. The electrostatic forces were generated by applying an electrical field using a DC voltage source of 24 kV and setting 15 cm between the nozzle tip and a flat aluminum foil, utilized as a collector. The spinning rates were regulated using a syringe pump that flowed out the solution from the metallic nozzle, which had a diameter of 0.9 mm. The production of mats with a homogeneous thickness of ~20 µm was promoted by a sliding system that moved the syringe pump from left to right at a speed of 20 mm/s. PPeF and PBF mats with good microstructural quality were obtained under the optimized processing conditions described in our previous paper [19]: the optimal PPeF concentration was 0.2 g/mL in a solvent mixture of DMF and CHCl_3_ added at a relative weight ratio of 1:5, applying a flow rate of 0.1 mL/min. For PBF, the optimal processing conditions were obtained with a concentration of 0.11 g/mL in a solvent mixture of HFIP:CHCl_3_ in a 1:1 volume ratio and a flow rate of 0.01 mL/min. The properties of these optimized solutions, including the viscosity, are reported in our previous work [19]. The optimized electrospinning parameters are listed in Table 1.

**Post-processing treatment of PBF and PPeF electrospun mats**. The list of the prepared electrospun mats with the applied post-processing parameters is summarized in Table 2. The mats were treated under vacuum and dried for 3 days at room temperature to remove the residual solvent that could affect their thermal and mechanical performance and electrochemical stability. The as-produced samples were referred to as PBF-N and PPeF-N (“neat”), whereas the samples treated under vacuum for 3 days were called PBF-T and PPeF-T (“treated”).

### 2.3. Characterization

The characterization reported here focuses on the main microstructural, thermomechanical, and functional properties that are fundamental for the target application (battery separators), while a detailed characterization of the mechanical behavior and the thermal stability of the membranes was reported in a previous work of our group [20].

**Microstructural characterization.** The microstructural features of the electrospun mats were investigated using a Zeiss Supra 60 (Carl Zeiss AG, Oberkochen, Germany) field-emission microscope (FESEM) operating at an acceleration potential of 2.5–3.5 kV. Before the observations, the samples were sputtered with a platinum/palladium coating for 20 s to render them conductive. The FESEM images were analyzed using Image J^®^ 1.53e software to determine the fiber diameter distribution and porosity of the electrospun mats.

**Porosity and pore distribution.** Disks with a diameter of 1 cm were die-cut for each sample (PBF-N, PBF-T, PPeF-N and PPeF-T), and the porosity was evaluated by considering the superficial pore distribution and calculated by dividing the surface pore area by the total surface area.

**Thermal properties.** The thermal properties of the prepared electrospun mats were evaluated through differential scanning calorimetry (DSC), performed with a Mettler DSC 30 calorimeter (Mettler Toledo, Inc., Columbus, OH, USA). Approximately 4 mg of PBF-N or PPeF-N mat was placed in an aluminum pan with a capacity of 40 µL and subjected to the following thermal program (heating/cooling rate = 10 °C/min): first heating scan from −50 °C to 250 °C, cooling scan from 250 °C to −50 °C, second heating scan from −50 °C to 250 °C under a nitrogen flow of 100 mL/min. The glass transition temperature (T_g_) of both polymers was calculated as the midpoint of the glass-to-rubber transition inflection step. Moreover, the melting temperature (T_m_), cold crystallization temperature (T_cc_), and corresponding specific enthalpy (Δ*H_m_*, Δ*H_cc_*) values were determined. The crystallinity degree (χ) of PBF was calculated using Equation (1):

(1) $$\chi =\frac{\Delta H_{m}-\Delta H_{cc}}{\Delta H_{0}}\times 100$$

where Δ*H*_0_ is the theoretical melting enthalpy of fully crystalline PBF (129 J/g) [24]. One specimen was tested for each composition.

**Chemical investigation.** Electrospun mats, with a thickness of 21.0 ± 1.0 µm and 25.0 ± 0.5 µm for PBF and PPeF, respectively, were preliminary evaluated with a solubility test to verify the stability of the membranes within the EC:DMC solution. PBF and PPeF disks were immersed in 5 mL of EC:DMC at a 1:1 wt%, using a typical solvent system for battery electrolytes, and the evolution shortly after soaking (5 min) and after 24 h was recorded. Given the solubility of PPeF in EC-DMC, the retention after 24 h of soaking in EC:DMC at 1:1 wt% was calculated for PBF samples using Equation (2):

(2) $$Electrolyte\;uptake\;\left(\%\right)=\frac{W_{wet}-{Wx}_{dry}}{W_{dry}}\times 100$$

The same test was performed on a Celgard 2300, a tri-layer polypropylene/polyethylene/polypropylene membrane, to compare the values with a commercial separator.

**Electrochemical characterization.** PBF and PPeF electrospun membranes with a thickness of 22.0 ± 3.0 µm were stored in a fridge at a constant temperature of 4 °C prior to electrochemical characterization. Polymeric disks with a 10 mm diameter (area = 0.785 cm^2^) were cut and dried at room temperature under vacuum conditions using a Buchi glass oven (B-585, Buchi Labortechnik AG, Flawil, Switzerland) for 16 h prior to electrochemical characterization. AC electrodes were prepared with 95 wt% activated carbon, 5 wt% conductive additive, and 5 wt% polytetrafluoroethylene (PTFE) from aqueous suspension by grinding the solids in a mortar and adding 100 µL of ethanol stepwise. Self-standing electrodes were laminated until a uniform 180.0 ± 5.0 µm thickness was obtained. The electrodes (9 mm diameter, 5–7 mg/cm^2^) were punched and dried in a vacuum at 120 °C overnight in a Buchi glass oven. Commercial electrode tapes based on lithium iron phosphate were also used for the characterization. These electrodes were cut (9 mm diameter, 6.5 mg/cm^2^) and dried using the same procedure as for the AC electrodes. The selected electrolyte solution was LP30. T-shaped Teflon cells (Bola, Bohlender GmbH, Grünsfeld, Germany) coupled with stainless steel plugs as current collectors were used to assess the electrochemical behavior of the electrospun membranes.

Electrochemical impedance spectroscopy (EIS), cyclic voltammetry (CV), and galvanostatic charge and discharge (GCD) measurements were performed using a VSP potentiostat/galvanostat (Biologic SAS, Seyssinet-Pariset, France) by EC-Lab 10.44 software. EIS tests were performed with a sinus amplitude of ±5 mV, 20 points per decade, and in a frequency range of 200 kHz–1 Hz to evaluate the contribution of the PBF membranes to cell resistance through the MacMullin parameter (*N_M_*), calculated using Equation (3):

(3) $$N_{M}=\frac{\sigma_{electrolyte\;}}{\sigma_{separator}}$$

where *σ_electrolyte_* was taken as 12.1 mS/cm [25], and *σ_separator_*, i.e., the conductivity of the separator soaked with the electrolyte, was calculated using Equation (4), knowing the thickness (*t_separator_*) and area (*a_separator_*) of the membrane and obtaining the ionic resistance (*r_ion_*) from the fitting of the EIS:

(4) $$\sigma_{separator}=\frac{t_{separator}}{r_{ion}\;\times \;a_{separator}}$$

To assess the electrochemical stability window (ESW) of the PBF membranes, CVs were performed with a scan rate of 20 mV/s for 25 cycles from 0.05 to 5 V using a stainless-steel current collector as the working electrode, an AC electrode with a 9 mm diameter as the counter electrode, and a lithium metal disk as a reference electrode.

## 3. Results and Discussion

**Figure 3:**
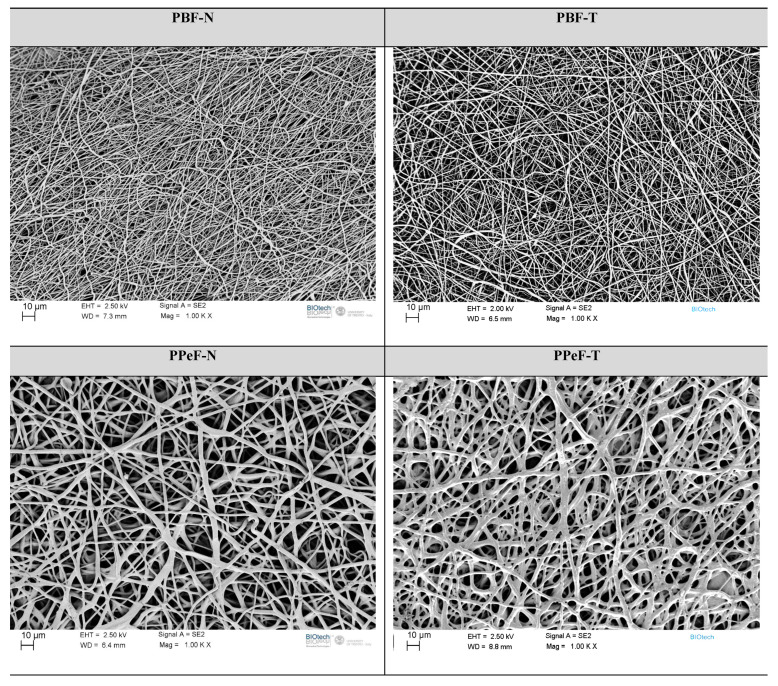
FESEM images of PBF and PPeF electrospun mats.

The microstructure of the PBF and PPeF electrospun mats is highlighted by the FESEM images reported in Figure 3. PBF-N and PBF-T have a homogeneous fiber distribution of 1.0 ± 0.2 µm and 1.1 ± 0.4 µm, respectively. The semicrystalline nature of PBF contributes to the stability of the membrane after solvent removal, and fibers maintain a rounded cross-section and smooth surface. PPeF-N and PPeF-T membranes show a heterogeneous distribution of partially fused and interconnected net of 2.6 ± 0.7 µm and 3.6 ± 0.9 µm, respectively, due to the amorphous nature of the polymer, with a T_g_ of 14 °C.

**Figure 4:**
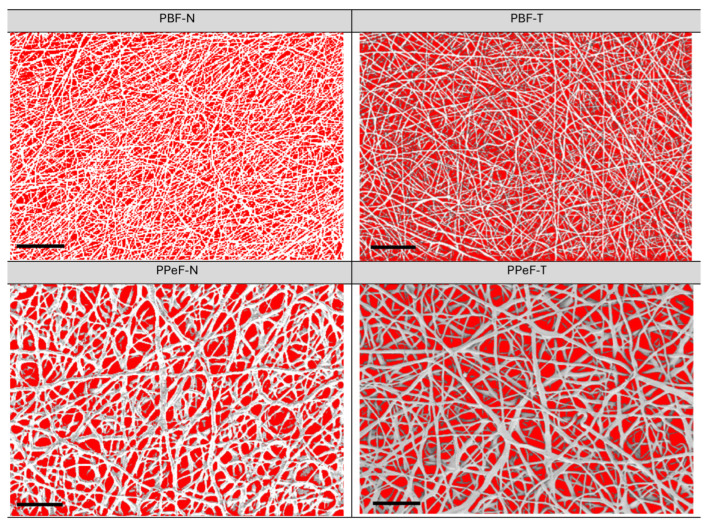
Graphical elaboration of the superficial porosity of PBF and PPeF (scale bar = 30 µm).

The porosity was acquired by ImageJ^®^ 1.53e software, which elaborated the voids of the electrospun mat in the FESEM images with red areas, as shown in Figure 4 and Table 3, enabling calculation of the superficial porosity as the pore area over the total area. PBF-N had a porosity of 53%, indicating a low degree of densification of the fibers that were homogeneously distributed, with an average fiber diameter of 1.0 ± 0.2 µm. PBF-T displayed a porosity of 54%, which was similar to the as-synthetized PBF-N, with a fiber distribution of 1.1 ± 0.4 µm, highlighting the stability of the membrane after the treatment. PPeF-N showed a porosity of 44%, indicating a higher degree of densification of the fibers. The FESEM image highlights a homogeneous distribution of the pores, with an average fiber diameter of 2.6 ± 0.7 µm and with junction between the fibers. The treatment for 3 days under vacuum conditions at room temperature affected the PPeF membrane porosity, which had a value of 32% and affected the fiber diameter, which increased to 3.6 ± 0.9 µm. Similar porosity values are typically obtained from electrospun monolayer separators, which have attracted increasing attention for enhancing the performance of LIBs thanks to their better lithium ion transportation and increased liquid electrolyte retention [26]. However, excessive porosity weakens the structure, leading to poor mechanical integrity. Ideally, the separator should have a sufficient but not excessively high porosity level to retain an adequate amount of electrolyte, so that it remains mechanically stable. This is the main feature that assures the safe operation of battery cells. Beyond the degree of porosity, the pore size and distribution, along with the fiber diameter, also affect the final properties of the separator [26].

**Figure 5:**
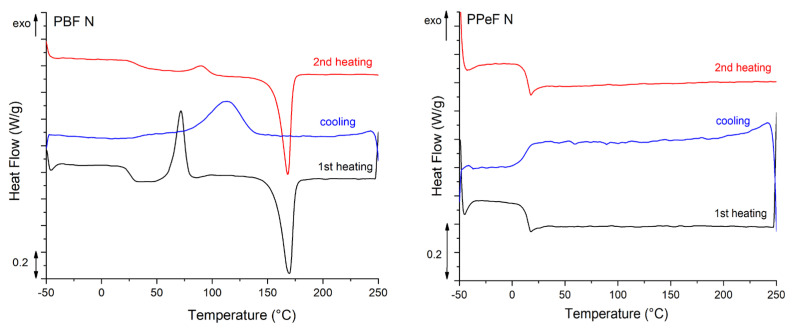
DSC thermograms of as-synthesized PBF and PPeF.

The main results of the DSC tests are reported in Figure 5 and Table 4. The semicrystalline nature of PBF is revealed by the DSC scans. In the first heating scan, PBF-N exhibits a distinct T_g_ of 25 °C, a pronounced cold crystallization peak at 71 °C, and a melting peak at 170 °C, resulting in a degree of crystallinity of 18.9%. The cooling scan of PBF highlights crystallization at 113 °C and a distinct shoulder at 47 °C, likely due to a solid-state arrangement. The second heating scan shows a higher T_g_ (31 °C), likely due to the evaporation of moisture and residual solvent, followed by a cold crystallization peak at 90 °C and a melting peak at 173 °C. Conversely, PPeF-N is fully amorphous, with a T_g_ at 14 °C. Indeed, the C-odd-numbered glycolic subunit impairs the capacity of PPeF to crystallize, thereby explaining the amorphous microstructure with the “oddeven effect” [17,18]. The relatively low T_g_ is explained by the presence of relatively long aliphatic subunits, which increase the molecular mobility. The preliminary DSC analysis highlights the fully amorphous structure of the as-spun PPeF mats that suffer operation conditions near the T_g_ more than the semicrystalline morphology of PBF, which is more suitable as a battery membrane due to its thermal stability.

Solubility tests of the electrospun membranes in EC:DMC solution were carried out as a preliminary investigation in order to evaluate their chemical stability before carrying out electrochemical tests in LP30, the conventional LIB electrolyte that contains EC and DMC as solvents. The results show that both the PPeF-N and PPeF-T disks dissolved after 5 min, while the PBF ones were still visible and retained their disk shape after 24 h.

Moreover, the solvent uptake measurements of the PBF samples were 531 ± 23 wt% and 658 ± 34 wt%, respectively, for PBF-N and PBF-T mats. These numbers align with the typical values reported in the literature for electrospun membranes [27,28,29,30]. As comparison, nonwoven polyolefin separators show electrolyte uptake values from 50% to 200%, depending on the polyolefin type and the electrolyte [31,32,33]. Usually, the desired values for an ideal membrane are around 50–150%. The membrane should act as a reservoir of electrolytes, providing and compensating possible electrolyte consumption and containing a larger amount of electrolyte to yield a higher ionic conductivity [30]. However, excessive electrolyte uptake might lead to the low energy density of the cell because of the additional weight and increased manufacturing costs due to the excess of electrolyte.

**Figure 6:**
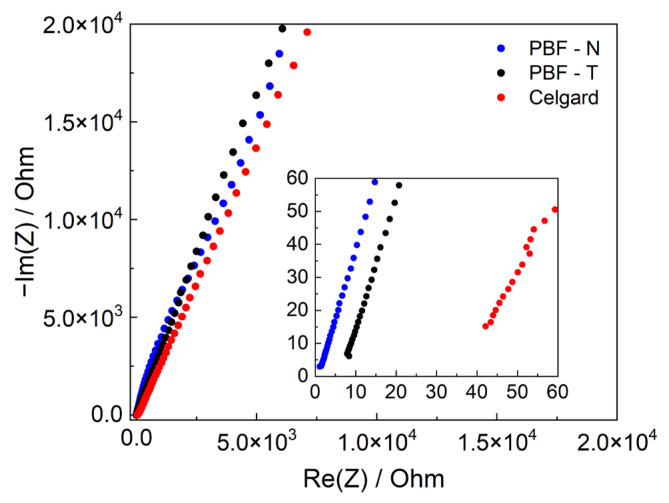
Electrochemical impedance spectra of symmetric stainless-steel cells in LP30 electrolyte with PBF-N (blue dots), PBF-T (black dots), and Celgard (red dots). The inset is a magnification of high-frequency data.

These preliminary analyses were essential for selecting PBF electrospun mats as the main candidates for electrochemical characterization, considering PPeF for future analysis as a membrane separator for aqueous electrolyte devices. The contribution of PBF membranes to the cell resistance through the MacMullin parameter (*N_M_*) was evaluated by EIS analysis, reported in Figure 6 and summarized in Table 5, the values of which were lower for the PBF samples than for those reported for Celgard. These lower values arise from the fact that electrospun membranes feature pores with a higher diameter and a decreased tortuosity, as also seen in the SEM images, which promote ionic mobility and higher ionic conductivity compared to nonwoven membranes.

**Figure 7:**
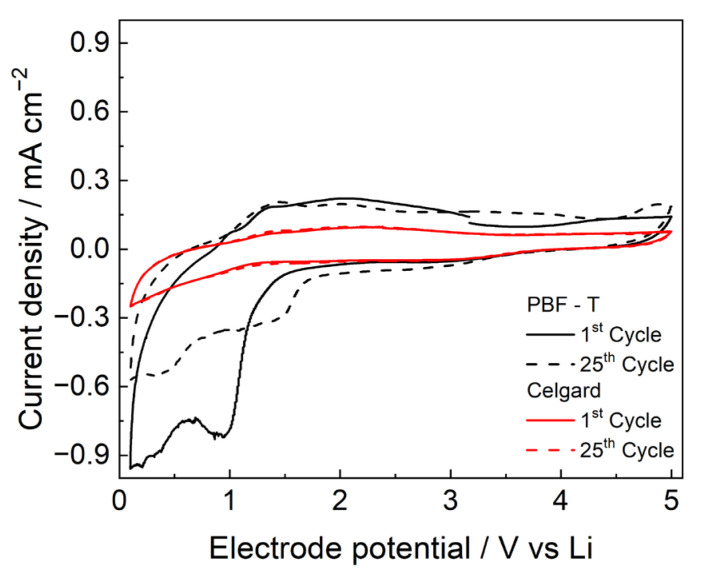
CVs performed at 20 mV/s in LP30 of PBF-T (black lines) and Celgard 2300 (red lines).

The CVs of the SS electrode in the presence of PBF-T separator, displayed in Figure 7, show box-shaped profiles in the potential range of 1.5 to 4.5 V, typical of capacitive behavior due to the AC counter-electrode and similar to that of Celgard 2300 in the same conditions. The membranes seem sufficiently stable up to 5 V, without the presence of Faradaic peaks during the cycles, making them compatible for use with high-voltage cathodes. However, PBF-T shows a reduction process in the first cycle around 1 V, which can be ascribed to the decomposition of some species that are closely related to polymer synthesis or to processes occurring at the surface of the stainless-steel electrode [34]. Current densities values decrease significantly after 25 cycles because the degradation products passivate the electrode, although a redox process is still visible. In the reverse scan between 1.2 and 3 V, some oxidation processes are visible, probably due to the reaction of the new species generated at the electrode [35].

**Figure 8:**
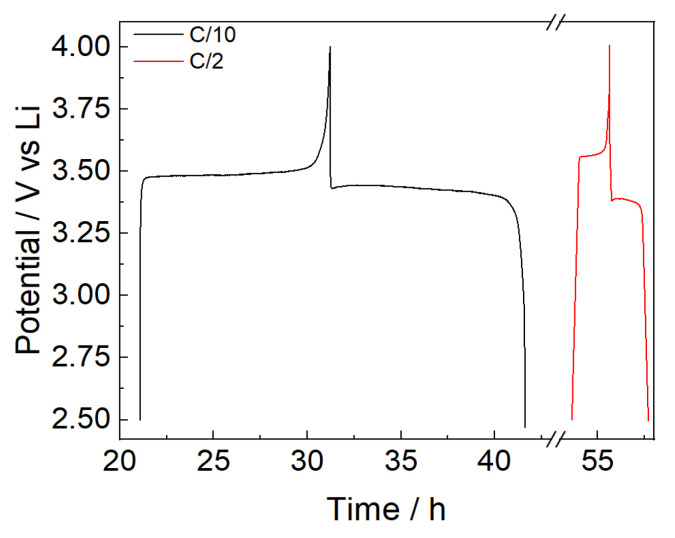
GCD cycles of an LP//LFP cell during GCD cycles at C/10 (black line) and C/2 (red line) in LP30.

The stability of the PBF-T separator was preliminary evaluated by GCD cycles of cells with LFP as the working and counter electrodes, using lithium metal as the reference electrode. GCD cycles were performed under constant-current charge and discharge between 2.5 and 4.0 V vs. Li. Before starting the GCD cycles, the working electrode was completely delithiated by using the reference electrode as the counter electrode in a two-electrode configuration. After delithiation, the cell FP//LFP was cycled in a three-electrode configuration. Three cycles at a C/10 rate were performed, followed by prolonged cycles at a C/2 rate. The charge and discharge voltage profiles for the two different C rates are displayed in Figure 8.

**Figure 9:**
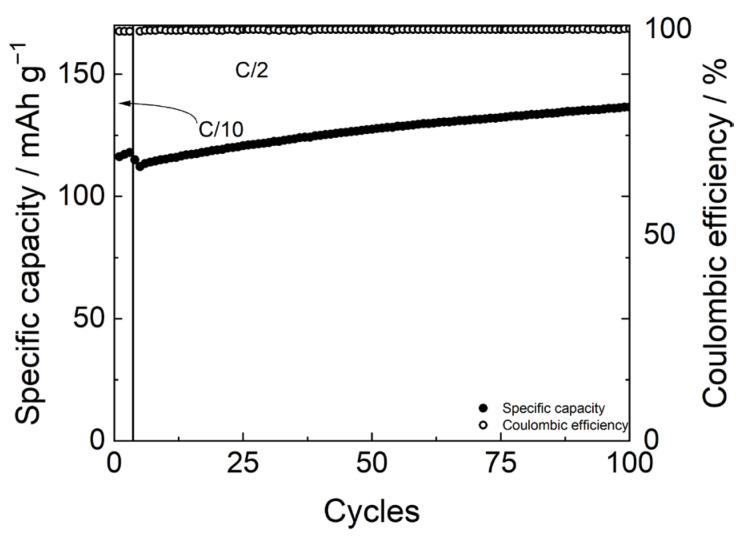
Specific capacity (full circles) and coulombic efficiency (plain circles ) of FP//LFP cell with LP30 electrolyte over cycling at C/2 after three formation cycles at C/10.

The potential profiles in Figure 8 show a slightly high overpotential, around 0.08 V at C/10 and 0.2 V at C/2. Nevertheless, Figure 9 shows the good stability of the FP//LFP cell with the PBF-T separator and LP30 electrolyte over cycling at a C/2 rate after three formation cycles at C/10. Due to the wettability of the membrane and the permeation of the electrolyte inside the electrodes [36,37], the specific capacity values are lower than the theoretical specific capacity (170 mAh/g) during the initial cycles both at C/10 and C/2, while the Coulombic efficiency values maintain constant close to 100%. Upon cycling, the wettability progressively improves, and after 100 cycles a specific capacity of 135 mAh/g is achieved, which seems interesting since these are values not far from what was obtained in other studies involving LFP electrodes coupled with a commercial separator [38].

## 4. Conclusions

PBF and PPeF are polymers derived from renewable sources and potentially suitable for use as separators in electrochemical energy storage devices. The electrospun PBF mat is characterized by good thermal and chemical stability, which make it suitable for application in LIBs. Given the high solubility of PPeF in the carbonate solvents used in conventional LIBs and SIBs, a possible use of PPeF could be in aqueous SIBs that have recently been under study given their increased sustainability and safety in comparison to the organic ones. The physicochemical characterization of PBF samples shows good MacMullin numbers and electrolyte uptake, with values better than those of conventional polyolefin membranes. Despite their porous nature currently preventing their use with lithium metal anodes, preliminary measurements in symmetric FP//LFP cells with LP30 electrolyte showed that these membranes exhibit good electrochemical stability, withstanding a hundred charge–discharge cycles. Further modifications to the electrospinning process could enable the use of these membranes, potentially allowing for more stable cycling in combination with other electrode materials.

## Figures and Tables

**Table 1:** Electrospinning process parameters for the production of PBF and PPeF mats.

Polymer	Solvent	Concentration (g/mL)	Flow Rate (mL/min)
PBF	H/C in ratio 1:1	0.11	0.01
PPeF	D/C in ratio 1:5	0.20	0.10

H = hexafluoro-2-propanol, C = chloroform, D = dimethylformamide.

**Table 2:** List of the prepared PBF and PPeF nonwoven mats with and without the post-processing parameters.

Label	Polymer	Concentration (g/mL)	Solvent	Flow Rate (mL/min)	Post-Processing
PBF-N	PBF	0.11	H/C in ratio 1:1	0.01	No
PBF-T	PBF	0.11	H/C in ratio 1:1	0.01	Yes
PPeF-N	PPeF	0.20	D/C in ratio 1:5	0.10	No
PPeF-T	PPeF	0.20	D/C in ratio 1:5	0.10	Yes

H = hexafluoro-2-propanol, C = chloroform, D = dimethylformamide.

**Table 3:** Porosity and fiber distribution of the prepared PBF and PPeF electrospun mats.

	PBF-N	PBF-T	PPeF-N	PPeF-T
Porosity (%)	53.6	54.5	44.9	32.4
Fiber diameter (µm)	1.0 ± 0.2	1.1 ± 0.4	2.6 ± 0.7	3.6 ± 0.9

**Table 4:** DSC data of as-synthesized PBF and PPeF.

		PBF-N	PPeF-N
1st heating scan	T_g_ (°C)	25.0	14.0
	T_cc_ (°C)	71.0	-
	Δ*H_cc_* (J/g)	32.2	-
	T_m_ (°C)	170.0	-
	Δ*H_m_* (J/g)	56.4	-
	χ (%)	18.8	-
Cooling scan	T_cc_ (°C)	113.0	-
	Δ*H_cc_* (J/g)	46.3	-
2nd heating scan	T_g_ (°C)	31.0	13.0
	T_cc_ (°C)	90.0	-
	Δ*H_cc_* (J/g)	5.2	-
	T_m_ (°C)	168.0	-
	Δ*H_m_* (J/g)	50.7	-

T_g_ = glass transition temperature, T_cc_ = cold crystallization temperature, Δ*H_cc_* = cold crystallization enthalpy, T_m_ = melting temperature, Δ*H_m_* = melting enthalpy, *χ* = degree of crystallinity.

**Table 5:** Parameters and conductivity of the separators soaked with LP30 electrolyte and MacMullin number (*N_M_*).

Separator	Area (cm^2^)	Thickness (cm)	*σ_separator_* (mS/cm)	*σ_electrolyte_* (mS/cm)	*N_M_*
PBF-N	0.785	2.2 × 10^−3^	4.0	12.1	3
PBF-T		2.0 × 10^−3^	1.2		10
Celgard		2.4 × 10^−3^	0.8		15

## Data Availability

The original contributions presented in this study are included in the article. Further inquiries can be directed to the corresponding author.

## Abbreviations

The following abbreviations are used in this manuscript:

PAFs	Poly(alkylene 2,5-furanoate)s
PBF	Poly(butylene 2,5-furandicarboxylate)
PPeF	Poly(pentamethylene 2,5-furandicarboxylate)
LIBs	Lithium-ion batteries
SIBs	Sodium-ion batteries
PE	Polyethylene
PP	Polypropylene
FDCA	2,5-furandicarboxylic acid
PEF	Poly(ethylene 2,5-furandicarboxylate)
PET	Poly(ethylene terephthalate)
PBT	Poly(butylene terephthalate)
2,5-DMF	Dimethyl 2,5-furandicarboxylate
1,4-BD	1,4-butanediol
1,5-BD	1,5-pentanediol
HFIP	Hexafluoro-2 propanol
DMF	Dimethylformamide
PTFE	Polytetrafluoroethylene
EC	Ethylene carbonate
DMC	Dimethyl carbonate
TBT	Titanium tetrabutoxide
TIP	Titanium isopropoxide
PMMA	Polymethylmethacrylate
FESEM	Field-emission scanning electron microscopy
DSC	Differential scanning calorimetry
EIS	Electrochemical impedance spectroscopy
CV	Cyclic voltammetry
GCD	Galvanostatic charge and discharge
ESW	Electrochemical stability window
LFP	Lithium iron phosphate
